# The Investigation of Compacting Cement Systems for Studying the Fundamental Process of Cement Gel Formation

**DOI:** 10.3390/gels8090530

**Published:** 2022-08-24

**Authors:** Alexey N. Beskopylny, Sergey A. Stel’makh, Evgenii M. Shcherban’, Levon R. Mailyan, Besarion Meskhi, Nikita Beskopylny, Diana El’shaeva, Maria Kotenko

**Affiliations:** 1 Department of Transport Systems, Faculty of Roads and Transport Systems, Don State Technical University, 344003 Rostov-on-Don, Russia; 2 Department of Engineering Geology, Bases, and Foundations, Don State Technical University, 344003 Rostov-on-Don, Russia; 3 Department of Roads, Don State Technical University, 344003 Rostov-on-Don, Russia; 4 Department of Life Safety and Environmental Protection, Faculty of Life Safety and Environmental Engineering, Don State Technical University, 344003 Rostov-on-Don, Russia; 5 Department Hardware and Software Engineering, Don State Technical University, 344003 Rostov-on-Don, Russia; 6 Department of Unique Buildings and Constructions Engineering, Don State Technical University, 344003 Rostov-on-Don, Russia

**Keywords:** gel, cement gel, sealing method, hardened cement paste, sealing cement systems, concrete structure

## Abstract

Fundamental knowledge of the processes of cement gel formation for new generation concretes is a scientific deficit. Studies aimed at the formation of a cement gel for standard vibrated concrete research, and especially for centrifugally compacted concrete, are of interest because the structure of this concrete differs significantly from the structure of standard vibrated concrete. This article aims to study the fundamental dependencies of the theoretical and practical values that occur during compaction using vibration, as well as the centrifugal force of new emerging concrete structures. New theoretical findings about the processes of cement gel formation for three technologies were developed: vibrating, centrifuging, and vibrocentrifuging of concrete; the fundamental difference in gel formation has been determined, the main physical and chemical processes were described, and a significant effect of technology on the gel formation process was established. The influence of indirect characteristics based on the processes of cement gel formation, rheological properties of concrete mixtures, water squeezing processes, and the ratio between the liquid and solid phases in the mixture was evaluated. The process of formation of cement gel for centrifugally compacted cement systems was studied and graphical dependences were constructed, giving answers to the mechanism of interaction according to the principle “composition-rheological characteristics-structure-properties of concrete”. The quantitative aspect of the achieved result is expressed in the increase in the indicators demonstrated by centrifuged and especially vibrocentrifuged samples compared to vibrated ones. Additionally, in terms of strength indicators, vibrocentrifuged samples demonstrated an increase from 22% to 32%, depending on the type of strength, and the rheological characteristics of concrete mixes differed by 80% and 300% in terms of delamination.

## 1. Introduction

### 1.1. Background

Fundamental knowledge about the processes of cement gel formation for new generation concretes is a scientific deficit. These ideas are not sufficiently developed in the existing scientific literature and are mainly based on theories of the hardening of binder cement systems and other components of the concrete mix, developed in the late 19th–early 20th century. Given the fact that the current construction industry receives more and more high-strength, structured concrete reaching classes above B100, a significant revision is required not only of engineering and technical parameters that are laid down when creating new knowledge about building materials, but also a revision of fundamental processes occurring not only at the macro but also at the micro level [1,2,3,4,5,6].

The influence of various factors on the formation of cement gel and the microstructure of the hardened cement paste are presented in Table 1.

Microstructural studies and modeling methods are especially important in studying the processes of cement gel formation and allow them to be controlled to improve and predict the characteristics of cement composites [22,23,24,25,26]. At the same time, the importance of the gel/space ratio for the compressive strength of cement composites is noted [27,28,29]. The study of the microstructure of calcium silicate hydrate (CSH) gels from theoretical and computational points of view, as well as the fractality of CSH and the evaluation of the mechanical properties and durability of CSH—with particular reference to carbonization and chloride/sulfate exposure—are also important in influencing the mechanical properties and durability of cement-based materials [30,31]. The most recent works of authors who studied the formation of the structure of cement pastes from the point of view of the mechanisms of gel formation and hardening are primarily aimed at the microstructural component of these processes and mechanisms [32,33,34,35,36]. Today, a large role is given to the study of the microstructure of cement pastes, in particular, microcracking [32], the characteristics of the pore structure [33], the effect of nanoadditives [34], and the mechanisms of carbonization and hydration, along with their products: calcium carbonate, aluminosilicate gel, and CSH phase [35]. The study of these processes and mechanisms at the micro level contributes greatly to developing an understanding of the gel-type contributions to various materials that form next-generation concretes [36].

### 1.2. Cement Gel Formation in Vibrated Compaction Concrete

As is known, in the initial period when cement is mixed with water, calcium hydroxide is released during the hydrolysis of tricalcium silicate, forming a supersaturated solution. This solution contains sulfate ions, alkali hydroxide, and a part of silica, alumina, and iron. A high concentration of calcium ions and sulfate ions is observed for a short time after mixing the cement with water since within a few minutes the first neoplasms—calcium hydroxide and ettringite—begin to precipitate from the solution.

Approximately an hour later, the second stage of hydration occurs, which is characterized by the formation of very fine calcium hydrosilicates. Since only the surface layers of cement grains take part in the reaction, the newly formed hydrated phases, called cement gel, are characterized by very fine granulometry and the cement grain size decreases slightly. Neoplasms first “appear on the surface of cement grains. With an increase in the number of new formations and their packing density, the boundary layer becomes less permeable to water for approximately 2–6 h” [37,38].

The second stage of delayed hydration is commonly referred to as the “latent or induction period” of cement hydration. During the latent period, the cement paste is a dense suspension, stabilized by the action of flocculation forces. However, the attractive forces between cement particles in water are relatively weak, which can be explained as follows: gel-coated cement grains form a solvate layer around themselves and have a positive E-potential. The combined action of the solvated layer and electric charge prevents direct contact between the grains in contact. At the same time, these grains experience interparticle attraction, at least in some boundary regions. The forces of repulsion and attraction are balanced at some distance from the interface, where the potential energy of the particles is minimal. Cement dough under the influence of these forces acquires coherence and mobility. During the latent period, there is a gradual absorption of water by the surface shells of cement grains; the thickness of the water layers between the grains decreases, and the mobility of the dough and concrete mixture gradually decreases. Osmotic pressure appears in the gel shells. The inner layers of cement grains, reacting with water, tend to expand. As a result, the destruction of the gel shells occurs, the access of water deep into the cement grains is facilitated, and the process of cement hydration is accelerated [37,38].

**Figure 1:**
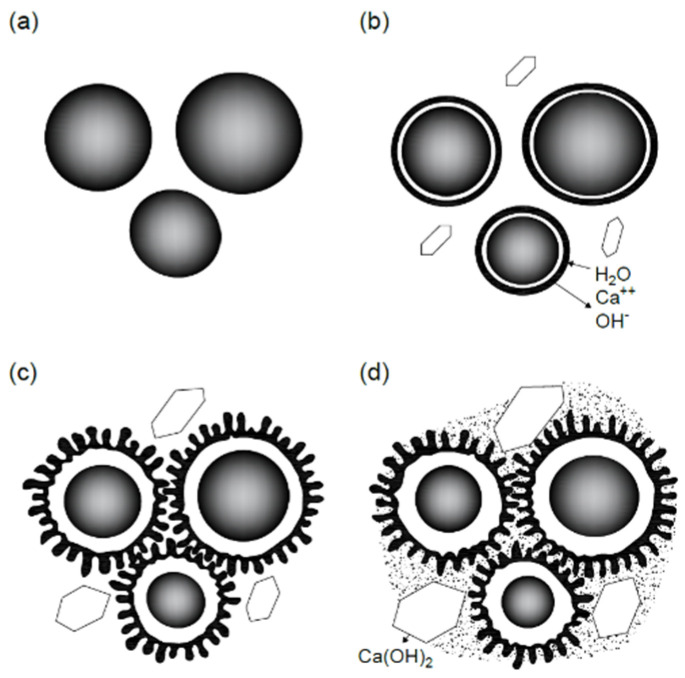
Scheme of the process of transformations in the structure of cement paste and hardened cement paste during cement hydration: (**a**) cement grains in water—the initial period of hydration; (**b**) formation of a gel shell on cement grains—a latent period of hydration; (**c**) secondary growth of the gel shell after the osmotic destruction of the original shell, the formation of wavy and columnar structures on the surface of the grains and in the pores of the hardened cement paste—the third period of hydration; (**d**) compaction of the structure of the hardened cement paste during subsequent hydration of the cement.

This is the third stage of the hydration process. It is characterized by the beginning of the crystallization of calcium hydroxide from a solution. This process is very intense. Since at this stage the number of hydrated phases is relatively small, in the space between the cement particles there is a free growth of thin plates of calcium hydroxide, calcium hydrosilicates, and ettringite in the form of long fibers that are formed simultaneously. The fibers of neoplasms pass through the pores, divide them into smaller ones and create a spatial bond, enhancing the adhesion between the hydrated phases and cement grains. With an increase in the content of hydrated phases, direct contacts arise between them, the number of which increases; the cement paste seizes and a hardened cement paste is formed. The resulting rigid structure is at first very loose, but gradually it becomes denser. New hydrate phases continuously appear in the water-filled pores of this structure. The volume of pores and their sizes decrease, the number of contacts between neoplasms increases, and the gel shells on the cement grains thicken and compact, coalescing into a continuous cement gel with inclusions of unreacted centers of cement grains. Schematically, the process of transformations occurring in the cement–water system during cement hydration is shown in Figure 1 [37,38].

### 1.3. Variotropic Structures of Concrete and Their Formation

Vibrated, centrifuged, and vibrocentrifuged concretes, including those in structures, differ in their structure, composition, and also in their characteristics [37,38,39,40,41,42,43,44,45,46]. Let’s consider the features and mechanisms of the processes occurring during each of these types of concrete compaction.

#### 1.3.1. Vibration

The most traditional way of compacting concrete in the formwork of a reinforced concrete product is by vibrating. When vibrating for a sufficiently long time, the larger and denser aggregate grains sink under the influence of vibration, while the smaller and lighter ones rise, thereby creating differences in the properties and characteristics of concrete along the section height, that is, leading to variatropy. Variatropy in vibrated concrete has the character of segregation, i.e., the separation of the concrete mixture in the process of vibrating and migration of heavy coarse aggregate and other heavy fractions to the lower parts of concrete structures. This segregation, which is the actual variability of concrete over the section, leads to a change in the properties and characteristics of concrete along the height of the section of a linear reinforced concrete element [37,38].

#### 1.3.2. Centrifugation

In centrifuged concrete, variatropy has a different character. Namely, it is expressed in the difference in the characteristics of concrete along the annular section. Variatropia here arises due to the centrifugal compaction of the concrete mixture. Heavy fractions of aggregate and other concrete components migrate to the outer part of the element, and lighter fractions to the inner part of the element. However, such variatropy is more positive than variatropy during vibration as the outer layer over the entire section of the element acquires the strongest characteristics of concrete, which carries the main load [39,40,41,42,43,44,45].

#### 1.3.3. Vibrocentrifugation

The most interesting, from our point of view, are vibrocentrifuged concretes in which variatropy is the result of the combined action of vibrating and centrifuging and is most pronounced. In such structures, with the strongest and most durable outer layer, the middle layer also has sufficient strength and can bear part of the load. The inner layer here also remains the weakest; however, the enhanced variatropy and the joint operation of the two bearing layers—the outer and middle ones—compensate for the weakness of the inner layer and, in total, lead to an even greater positive effect in the calculation, design, and operation of such structures [40,41,42,43,45,46].

### 1.4. Rationale

The process of forming a cement gel is fundamental to the hardening of concrete, and directly affects the formation of the structure and properties of such concrete. Of course, one of the key roles in this process is played by the composition of concrete. However, our study is not devoted to the influence of the composition, but rather to the concrete mixture compaction technology. This is because technological parameters are no less important at the stage of creating new concrete structures, and these technological parameters in turn depend on fundamental physical and chemical processes that can be controlled when forming new types of material structures. In this regard, it will be interesting to study the formation of a cement gel for standard vibrated concretes, especially for centrifugally compacted concretes, since the structure of such concretes will differ significantly from the structure of standard vibrated concretes. First, this is due to the influence of fundamental centrifugal forces, which create conditions for a fundamentally different process of hydration and the formation of a cement gel by squeezing out the liquid phase and a significant separation between the liquid and solid components in concretes [39,40]. Of course, the process of cement gel formation, which is more fundamental, less studied, and accordingly less controllable, will differ significantly in this regard.

Thus, the main goal of our study is to study the fundamental dependencies of theoretical and practical quantities that arise during compaction with the help of vibration, as well as the centrifugal force of new emerging concrete structures.

The objectives of the study will include a detailed literary analysis of existing ideas that are already quite outdated, their development on the example of more advanced concrete structures with higher rates, and finally, the application of the obtained fundamental dependencies in the practical construction industry to establish the relationship between the fundamental processes of cement formation gel using various technologies, and an applied aspect expressed as an increase in the performance of hardened concretes used in modern construction.

**Figure 2:**
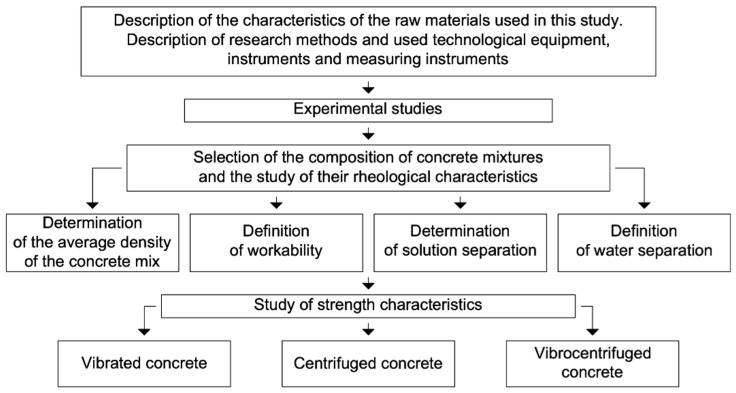
Plan of experimental studies.

The experimental research plan is presented in the form of a diagram in Figure 2.

## 2. Results

### 2.1. The Results of the Selection of the Composition of Concrete Mixtures and the Study of Their Characteristics

For the manufacture of concrete using the technologies of vibration, centrifugation, and vibrocentrifugation; the compositions (Table 2) were selected with the grade of concrete mix for workability P1 (cone draft 1–4 cm) and the design class of concrete B30.

Table 3 presents the results of determining the rheological characteristics of the manufactured concrete mixes.

According to the results of studies of the rheological characteristics of concrete mixtures, it was found that the density of a centrifugally compacted concrete mixture is up to 7% higher, the solution separation is 80% less, and the water separation is three times less than that of a vibrated mixture.

### 2.2. Study of the Physical and Mechanical Characteristics of Concrete

The results of experimental studies of the physical and mechanical characteristics of vibrated concrete are presented in Table 4.

**Figure 3:**
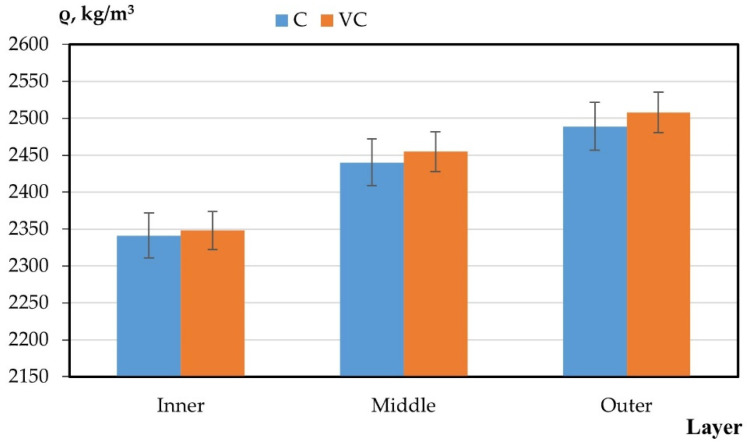
Change in density by layers (C—centrifuged concrete, VC—vibrocentrifuged concrete).

**Figure 4:**
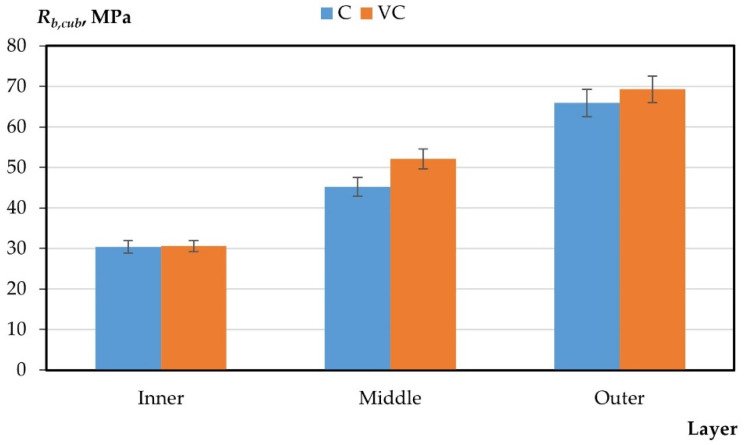
Change in compressive strength by layers (C—centrifuged concrete, VC—vibrocentrifuged concrete).

**Figure 5:**
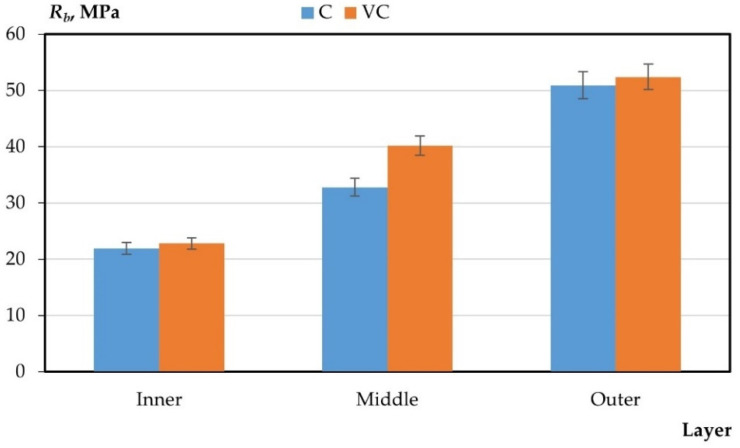
Change in axial compressive strength by layers (C—centrifuged concrete, VC—vibrocentrifuged concrete).

**Figure 6:**
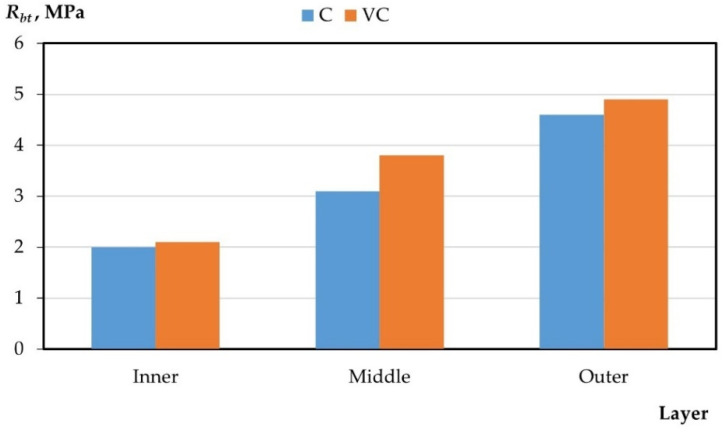
Change in axial tensile strength by layers (C—centrifuged concrete, VC—vibrocentrifuged concrete).

**Figure 7:**
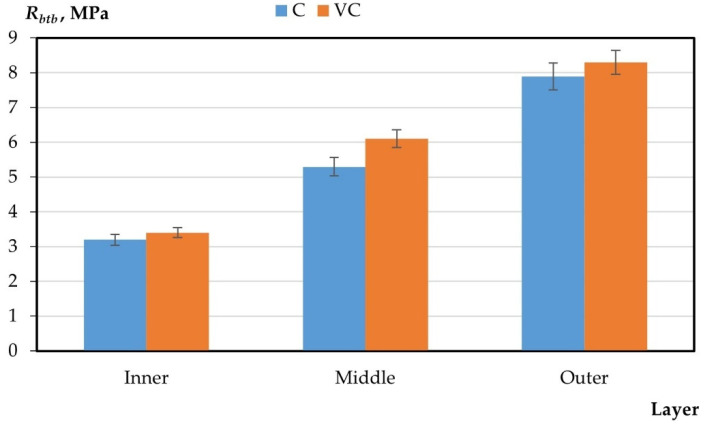
Change in tensile strength in bending by layers (C—centrifuged concrete, VC—vibrocentrifuged concrete).

The results of the experimental studies on the integral and differential characteristics of the layers of variotropic sections of centrifuged and vibrocentrifuged concrete samples are presented in Table 5 and Figure 3, Figure 4, Figure 5, Figure 6 and Figure 7, respectively.

From the results presented in Table 4 and Table 5 and in Figure 3, Figure 4, Figure 5, Figure 6 and Figure 7, it can be seen that vibrocentrifuged concrete has the best physical and mechanical characteristics, both integral in comparison to vibrated and centrifuged, and differential in comparison to centrifuged. At the same time, the characteristics of the inner and outer layers are practically the same for vibrocentrifuged and centrifuged concrete. The main advantage of vibrocentrifuged concrete over centrifuged concrete is the hardened middle layer.

### 2.3. Study of the Microstructure of Concrete

**Figure 8:**
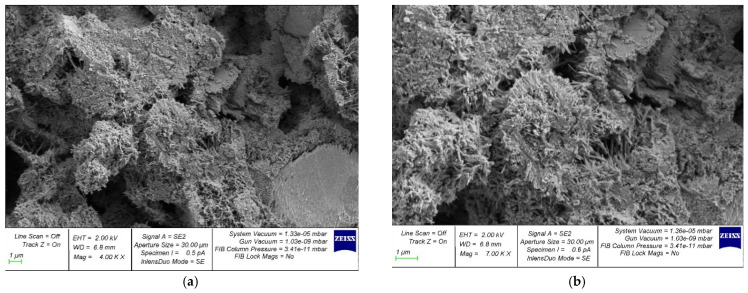
Photo of samples of centrifuged hardened cement paste: (**a**) at 4000× magnification; (**b**) at 7000× magnification.

**Figure 9:**
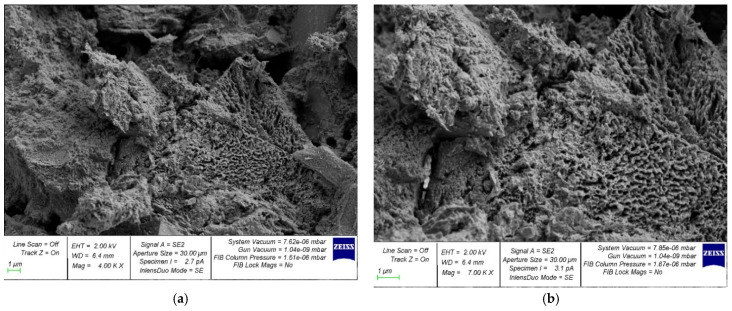
Photo of vibrocentrifuged hardened cement paste samples: (**a**) at 4000× magnification; (**b**) at 7000× magnification.

Figure 8 and Figure 9 show photographs of the microstructure of centrifuged and vibrocentrifuged hardened cement paste.

Comparing Figure 8a and Figure 9a, one can see the difference in the structure of the centrifuged hardened cement paste from the vibrocentrifuged one. In particular, the centrifuged sample (Figure 8a) shows large voids and pores. The vibrocentrifuged sample (Figure 9a) has far fewer voids and pores, and they are noticeably smaller, which indicates a more solid and organized structure compared to the centrifuged sample. This also confirms the above results of physical and mechanical testing of concrete.

In addition, Figure 8b and Figure 9b show that the vibrocentrifuged hardened paste sample (Figure 9b) has a more solid structure with fewer and smaller filtration channels than the centrifuged sample (Figure 8b). A large difference in the density of the cement gel over the thickness of the concrete section is exacerbated by the presence of directed radial filtration channels, the cross-section and number of which increase from the outer to the inner surface of the element [39,40]. With vibrocentrifugation, their number and size are much smaller than with centrifugation, which helps to improve the physical and mechanical characteristics of concrete and products made from it. The SEM analysis performed confirms this and is in good agreement with [39,40].

## 3. Discussion

According to the results of experimental studies on the mechanical characteristics of centrifuged and vibrocentrifuged concrete, it can be concluded that the integral strength characteristics of vibrocentrifuged concrete are higher than those of centrifuged (by 7–18%) and vibrated concrete (by 22–32%), depending on the type of strength. At the same time, centrifuged concrete has better strength indicators than vibrated concrete. The same trend is typical for density.

If we compare the differences in the values of the strength characteristics of layers of centrifuged and vibrocentrifuged concrete, then between the middle and inner layers, the difference is greater for vibrocentrifuged (70–81%) than for centrifuged (48–65%). While the difference between the outer and middle layers of vibrocentrifuged concrete is less (29–36%) than that of centrifuged concrete (45–55%), the differences in strength characteristics between the outer and inner layers of vibrocentrifuged and centrifuged concrete are approximately the same and differs only by a few percent. This indicates the strengthening of the middle layer of vibrocentrifuged concrete in comparison with centrifuged concrete.

The differences in the values of the integral strength characteristics of vibrated, centrifuged and vibrocentrifuged concretes and the differential characteristics of centrifuged and vibrocentrifuged concretes are summarized in Table 6 and Table 7, respectively.

The calculation results presented in Table 6 and Table 7 numerically confirm the conclusions drawn from the results of the experimental studies.

The analysis of the microstructure of the samples is in good agreement with the results of physical and mechanical tests of concrete, confirming the better properties and structure of vibrocentrifuged concrete compared to centrifuged concrete, including in terms of cement gel formation. An analysis of the microstructure also allows us to speak about the improvement of the properties that characterize the durability of vibrocentrifuged concrete in comparison with centrifuged and especially vibrated concrete, due to a decrease in the number and size of filtration channels, pores, and voids, leading to compaction and strengthening of the structure, and a decrease in concrete permeability [45].

To highlight the essence of centrifugal molding and compaction it is necessary to understand when a mold with a uniformly distributed concrete mixture in it rotates around a fixed axis at a certain speed, a compressing centrifugal pressure arises, under the influence of which a liquid with finely dispersed fractions is squeezed out of the cement gel and larger particles approach each other of the solid phase. As a result of this process, the cohesion of the cement gel increases, and the compaction of the concrete mixture is achieved. The “ability of the concrete mixture to be evenly distributed over the inner surface of the rotating mold is determined by the viscoplastic properties of the cement gel. If you place a viscoplastic or short-term vibrationally liquefied concrete mixture into a rotating mold, then under the action of centrifugal radially directed pressure, it will begin to deform - spread in all directions. This will continue until there is an equilibrium between the forces that promote the distribution of the mixture in the mold and the structural compressive strength of the cement gel. With an increase in the rotation speed of the mold, the liquid phase will begin to be squeezed out of the cement gel and this will continue until the compacted concrete mixture acquires the properties of a pseudo-solid body: the deformation of which can manifest itself mainly in the form of an elastic aftereffect” [39,40].

The “centrifugal pressure is directly perceived by the liquid phase, in connection with which the excess hydrostatic pressure increases, under the influence of which the liquid is filtered and at the same time the concrete mixture is compacted”. In addition, the compressibility of the “cement gel decreases and an equilibrium state” is established, characterized by an internal redistribution of pressure and termination of the filtration process due to significant resistance to the movement of fluid through the pore channels. Pressing pressure, like hydrostatic pressure, varies unevenly across the wall thickness of the product: from a minimum on the inner to a maximum on the outer surface of the product, so the liquid phase cannot be simultaneously squeezed out of the entire thickness of the concrete. First, the liquid is squeezed out more completely from the outer layers of concrete, where the greatest pressing pressures act, and as it approaches the inner surface of the element less and less liquid phase is displaced. From the inner layer, where the pressing pressure is close to zero, the liquid does not separate at all since this is also prevented by the layer of previously squeezed-out liquid. It follows that the mechanism of water separation from the concrete mixture is a hydrodynamic process of fluid filtration from one layer of concrete to another, continuing until all excess fluid is squeezed out of the product and a new balance between internal and external forces is restored. Therefore, different layers of concrete will contain different amounts of the liquid phase. Significant uneven density of centrifugation of the cement gel over the thickness of the product is exacerbated by the presence of directed radial filtration channels, the cross-section and number of which increases from the outer to the inner surface of the element.

The cross-section and the number of filtration channels depend on the initial water content in the cement gel: the less free liquid in it, the less it will be squeezed out during centrifugation and the filtration channels will be more dispersed. They may not occur at all if the liquid phase is not squeezed out. This is possible, for example, when vibration and centrifugation are combined in time. With layer-by-layer centrifugation, the number and cross-section of filtration channels are significantly reduced, along with their outlets in each compacted layer of cement gel overlap. If the squeezed liquid is removed after compaction of the first layer, then during compaction of the second layer it will exfoliate mainly only from it. At the same time, additional compaction of the first layer will occur (under the influence of increased pressing pressure from the mass of the two layers) and due to the deformation of the filtration channels, a certain amount of liquid will be displaced from them. Since before the distribution of the second layer, most of the liquid was removed from the first layer, during the compaction of the second layer, a less developed system of filtration channels is formed in it than when both layers are centrifuged simultaneously. The third, fourth, and other layers of cement gel will be compacted similarly. As a result of layer-by-layer compaction, the difference in values (W/C) of the individual layers of cement gel will decrease. With layer-by-layer centrifugation, a less porous (by 10–12%) structure of the hardened cement paste is formed than with single-layer centrifugation. In addition, with a decrease in porosity the pore structure changes, which significantly increases the water resistance of centrifuged concrete [39,40].

The most important physical parameter determining the duration of the involvement of elementary layers of cement gel in the compaction process and the end of the liquid phase exfoliation at a given pressing pressure is the time of centrifugation of the concrete mixture. As the centrifugation time increases the water separation process continues, but the strength of concrete, as a rule, decreases.

The pressing pressure has a similar effect: the greater it is, the faster the liquid phase separates in the initial period; therefore, with an increase in the time of centrifugation beyond the optimal duration, the strength of concrete decreases. The natural decline in the strength of concrete at high values of pressing pressure after some optimal compaction time of the mixture is caused by a change in the properties of the structure of the cement gel during its severe dehydration. With severe dehydration of cement gel in compacted concrete, tensile stresses may occur, leading to the opening of radial cracks in the wall of the product. The crack opening is significantly affected by the elastic aftereffect of the steel split mold and partly by the uneven distribution of centrifugal pressure on the concrete mixture [39,40].

The uneven density of centrifuged concrete is expressed not only in the structural heterogeneity of the hardened cement paste but also in the nature of the distribution of filler grains along the wall thickness of the product. The larger grains are pressed against the outer surface of the product, and the smaller ones are concentrated closer to its inner surface, so the centrifuged concrete differs from the vibrated one by the heterogeneous distribution of filler grains along the thickness of the product. With a properly selected composition of concrete, a 0.5–2 mm thick sludge crust forms on the inner surface of the element wall, followed by a layer of hardened cement paste, then fine-grained concrete and concrete of ordinary texture. Following this, the distribution of cement along the section of the wall of the product also changes. Such heterogeneity reduces the strength of the centrifuged concrete compared to what it should have been with a uniform distribution of filler grains over the entire section of the element. In concrete, the cement gel fills only the intergranular space, occupying about 1/3 of its volume, so the filtration ducts formed in the cement gel are dispersed and change their direction when they meet the filler grains. In this regard, the length and type of filtration ducts change. Skirting the contact surface between the cement gel and the filler grains, the filtration ducts merge with the solvate layer on the surface of the filler grains and then the ducts form again, breaking through the cement gel layers. This is repeated until the pressed liquid comes out on the inner surface of the product. Consequently, during the centrifugation of the concrete mixture, the liquid is also squeezed out through filtration channels in a cement gel.

The main disadvantage of the centrifugal method is the separation of the liquid phase (sludge) and the uneven distribution of components in concrete. The formed filtration channels during single-layer centrifugation significantly increase the permeability of concrete. An additional measure to reduce cement consumption and sludge yield is the use of vibration centrifugation since in this case very little liquid phase is squeezed out and less centrifugal pressure can be used to seal the concrete mixture. If centrifugation is combined with horizontally directed high-frequency vibration, then the initial water content in the cement gel can be reduced and compaction will occur without sludge detachment due to the redistribution of the liquid phase and other known factors [39,40].

## 4. Conclusions

(1) The processes of cement gel formation for three technologies were studied: vibrating, centrifuging, and vibrocentrifuging of concrete. The fundamental difference in gel formation was determined, the main physical and chemical processes were described, and a significant effect of technology on the gel formation process was established.
(2) The influence of indirect characteristics based on the processes of cement gel formation, the rheological properties of concrete mixtures, the processes of squeezing water, and the ratio between the liquid and solid phases in the mixture was evaluated.
(3) The formation of cement gel for centrifugally compacted cement systems has been studied, and graphical dependencies have been constructed, giving answers to the mechanism of interaction according to the principle “composition-rheological characteristics-structure-properties of concrete”.
(4) The quantitative aspect of the result achieved is expressed in the increase in performance demonstrated by centrifuged and especially vibrocentrifuged samples compared to vibrating ones. The rheological characteristics of concrete mixes differed by 80% and 300% in terms of delamination. In terms of strength indicators, vibrocentrifuged samples showed an increase from 22% to 32% compared to those of vibrating and from 7% to 18% compared to those centrifuged, depending on the type of strength. The difference between the middle and inner layers is greater for vibrocentrifuged concrete (70–81%) than for centrifuged concrete (48–65%). The difference between the outer and middle layers of vibrocentrifuged concrete is less (29–36%) than that of centrifuged concrete (45–55%). This indicates the strengthening of the middle layer of vibrocentrifuged concrete in comparison with centrifuged concrete.
(5) Analysis of the microstructure showed that the vibrocentrifuged hardened cement paste has a more solid structure with a smaller number and size of voids, pores, and filtration channels than centrifuged.

Prospects for the development of the study are seen in the continuation and deepening of the study of the processes of cement gel formation for other compaction technologies, particularly vibrohydraulic pressing, which make it possible to obtain fundamentally different rheological characteristics of the concrete mixture, the processes occurring at the “liquid-solid” phase boundary, and the final impact of the ongoing fundamental processes on applied properties of the resulting concretes with the creation of an empirical and theoretical basis for the practical industry.

## 5. Materials and Methods

### 5.1. Materials

The main characteristics of Portland cement CEM I 52.5 (Novoroscement, Novorossiysk, Russia) are presented in Table 8.

The used quartz sand produced by Arkhipovsky Quarry OJSC (Arkhipovskoye village, Russia) had the following characteristics: “fineness modulus—1.66; the content of dust and clay particles is 1.2%; bulk density—1438 kg/m^3^; true density—2650 kg/m^3^”.

The used granite crushed stone produced by Pavlovsknerud JSC (Pavlovsk, Russia) had the following characteristics: “fraction size—5–20 mm; bulk density—1503 kg/m^3^; true density—2620 kg/m^3^; crushability—11.4% by weight; the content of lamellar (flaky) and acicular grains is 8.1% by mass; voidness—43%”.

### 5.2. Methods

Selection of the composition of the concrete mixture for vibrated (V) concrete in accordance with the requirements of GOST 27006 “Concretes. Rules for mix proposing” [47] include:

- appointment of a nominal composition based on the calculation, experimental verification, and adjustment of the initial compositions;
- appointment of the working composition of concrete;
- adjustment of the working composition of concrete upon receipt of batches of materials of the same types, grades, and classes that were taken in the selection of the nominal composition, but taking into account their actual quality;
- checking in laboratory and production conditions of working compositions after their correction [47].

The selection of the composition of the concrete mixture for the manufacture of centrifuged (C) and vibrocentrifuged (VC) concretes was carried out in accordance with the recommendations of VSN-1-90 “Fabrication Requirements for Centrifugal-Cast Support Pillars for Contact Lines, Communications Lines, and Lock-Out Systems”.

The approximate value of the required residual water–cement ratio in the calculation and selection of the composition of centrifuged concrete was determined by the formula

(1) $${\left(W/C\right)}_{res}=\frac{0.45\times R_{cem}}{R_{b}+0.18\times R_{cem}}$$

where *W* is the water content (residual) in 1 m^3^ of concrete, l; *C* is cement consumption per 1 m^3^ of concrete, kg; $R_{cem}$ is the brand or activity of the cement; $R_{b}$ is the compressive strength of concrete corresponding to the design class.

The required cement content per 1 m^3^ of concrete is calculated by the formula:

(2) $$C=\frac{W_{res}}{{\left(W/C\right)}_{res}}$$

where $W_{res}$ is the amount of residual water in concrete (after centrifugation), taken for approximate calculations equal to 135–150 L/m^3^ depending on the type of plasticizer used.

The absolute volume of the cement paste $V_{m}$, L, the absolute volume of the mixture of aggregates (sand + crushed stone) $V_{a}$, L, and the total weight consumption of aggregates *A* and kg, respectively, were determined by the formulas

(3) $$V_{m}=\frac{C}{\rho_{c}}+W$$

(4) $$V_{3}=1000-V_{m}$$

(5) $$A=V_{a}\times \rho_{a}$$

where $\rho_{c}$ is the density of cement, kg/L; $\rho_{a}$ is the reduced density of the mixture of sand and crushed stone, kg/L, determined by the formula:

(6) $$\rho_{a}=\frac{\rho_{cs}+\rho_{s}}{1+r}$$

Here $\rho_{s}$ and $\rho_{cs}$ are the density of sand and crushed stone, respectively, and kg/L; *r* is the mass ratio between sand and gravel.

The weight consumption of crushed stone *CS*, kg, sand, *S*, and kg was determined by the formulas

(7) $$CS=\frac{A}{1+r}$$

(8) S=A−CS 

The workability of concrete mixtures for the manufacture of concrete by various technologies was evaluated by the draft of the cone. The cone was installed on a smooth sheet and filled with concrete mixture through a funnel in three layers of the same height. Each layer was sealed by bayoneting with a metal rod 25 times. Then the cone was smoothly removed from the molded concrete mixture in a strictly vertical direction and installed next to it. Cone slump was determined by placing a smooth rod on top of the cone and measuring the distance from the lower surface of the rod to the surface of the GOST 10181 “Concrete mixtures. Methods of testing” [48].

The delamination of the concrete mixture was evaluated by indicators of solution separation and water separation. The mortar “separation of a concrete mixture with coarse aggregate, which characterizes its delamination under dynamic action, was determined by comparing the content of the mortar component in the lower and upper parts of the concrete mixture, compacted in a measuring vessel or form”. Determination of the solution separation of the concrete mixture was carried out as follows. The concrete mixture was poured into a measuring vessel, compacted, and vibrated for 25 s; “After additional vibrating, the top layer of the concrete mixture with a height of about half the height of the measuring vessel was taken onto a pre-weighed baking sheet, and the mixture remaining in the lower part of the measuring vessel (mould) was vibrated until the surface of the mixture was leveled”. Then, “with an error of up to 5 mm, the height of the layer of the mixture remaining in the lower part of the measuring vessel (mould) was measured, and the height of the selected layer of the mixture was calculated”. The mixture remaining in the mold was spread on a second weighed baking sheet. The concrete mixture “divided into two portions from the upper and lower parts of the measuring vessel (mould) was weighed and subjected to wet sieving on a sieve with holes 5 mm in diameter”. Additionally, “During wet sieving, each weighed portion of the mixture, laid out on a sieve, was washed with a stream of clean water until the cement-sand mortar was completely removed from the surface of coarse aggregate grains” [48].

The solution separation of the concrete mix *P_m_*, %, is determined by the formula:

(9) $$P_{m}=\frac{m_{m.up}-m_{m.low}}{m_{m.up}+m_{m.low}}\times 100$$

(10) $$m_{m.up}=\left(m_{mix.up}-m_{cs.up}\right)\times \frac{0.5H}{H_{up}}$$

(11) $$m_{m.low}=\left(m_{mix.low}-m_{cs.low}\right)\times \frac{0.5H}{H_{low}}$$

$m_{m.up},m_{m.low}$ are “the mass of the solution component of the mixture located in the upper and lower parts of the measuring vessel”, g; $m_{mix.up},m_{mix.low}$ are “the mass of the concrete mixture taken from the upper and lower parts of the measuring vessel (mould)”, g; $m_{cs.up},m_{cs.low}$ are “mass of dried coarse aggregate contained in samples from the upper and lower parts of the measuring vessel”, g; *H* is the “height of the measuring vessel”, mm; and $H_{up},H_{low}$ are the “actual heights of the upper and lower layers of the mixture”, respectively.

The water separation of the concrete mixture “was determined after settling it in a measuring vessel for a certain period of time. The concrete mixture was placed in a measuring vessel and compacted on a vibrating platform for 25 s, then the vessel was covered with a glass sheet and left alone for 2 h. Then, the separated water was taken every 15 min with a pipette, collecting it in a beaker with a lid, and weighing after the end of the test” [48].

The water separation of the concrete mixture $P_{ws}$, %, was characterized by the volume of water released from the concrete mixture in 2 h, related to the volume of the concrete mixture in the measuring vessel, and was calculated by the formula:

(12) $$P_{ws}=\frac{m_{ws}}{\rho_{b}\times V_{c.sm}}$$

where $m_{ws}$ is the “mass of separated water”, g; $\rho_{w}$ is the “density of water, taken equal to 1 g/cm^3^”; $V_{c.sm}$ is the “volume of compacted concrete mix” per cm^3^.

The average density of the concrete mixture $\rho_{av.m}$, kg/m^3^ was calculated by the formula:

(13) $$\rho_{av.m}=\frac{m-m_{1}}{V}\times 1000$$

where *m* is the mass of the measuring vessel with the concrete mixture, g; $m_{1}$ is the mass of the measuring vessel without mixture, g; *V* is the capacity of the measuring vessel in cm^3^.

Compressive strength, tensile strength in bending, and axial tensile strength were evaluated according to [49].

For the manufacture of centrifuged and vibrocentrifuged concretes, special laboratory facilities were used for the manufacture of samples of this type; schemes of these facilities are presented in [50,51]. The mode of molding experimental centrifuged and vibrocentrifuged concretes was described in detail earlier in [42].

The technique for manufacturing “prototypes for determining the strength integral and differential characteristics of concrete” is presented in [41,42]. The program for the manufacture of prototypes for determining “integral and differential indicators from centrifuged and vibrocentrifuged elements of an annular section with dimensions”: outer diameter 450 mm; hole inner diameter 150 mm; and total height 1200 mm, presented in [41]. The differential characteristics of centrifuged and vibrocentrifuged concretes were evaluated for three layers of an annular section: inner, middle, and outer.

Technological and testing equipment and measuring instruments were used the same as in [52,53,54,55].

## Figures and Tables

**Table 1:** Influence of various factors on the formation of cement gel.

Ref. Number	The Type of Factor Considered in the Work	Name of the Influencing Factor	The Influence of the Considered Factor on the Formation of Cement Gel and the Microstructure of the Hardened Cement Paste
[7]	Prescription (use of a nano-modifying additive	nano-SiO_2_	The addition of nanosilica promotes “an increase in the degree of hydration of the cement paste and a more active formation of the C-S-H gel, as well as a decrease in the overall porosity of the hardened cement paste” [7].
[8,9]	Prescription (use of an additive as a replacement for part of the cement)	bottom ash from power plants	Ash-based cement pastes contain more CSH than unadded cement pastes.
[10]	Prescription (use of an additive as a replacement for part of the cement)	low calcium fly ash	The loose and porous microstructure of fly ash results in “higher water consumption, which reduces the flowability of cement-based pastes. The main hydration products of cement-based materials mixed with LCFA were AFt, CSH gel and Ca(OH)_2_” [10]
[11,12]	Prescription (use of an additive as a replacement for part of the cement)	belite cement and fly ash	Hydration of belite cement from fly ash “promotes the formation of CSH gel, ettringite and calcium hydroxide, thereby greatly increasing long-term strength” [11], and also reduces the porosity of the hardened cement paste
[13]	Prescription (use of a nano-modifying additive)	carbon nanofibers	Carbon nanofibers fill nanopores and connect grains of calcium silicate hydrates (C–S–H), also nanofibers affect the probability distribution function of the local packing density, causing a shift towards higher values
[14,15]	Prescription (supplement use)	$Bi_{2}O_{3}$$\;\mathrm{and}\;ZrO_{2}$ radio insulators	Radiopaque agents increase the average length of the silicate chain and the degree of substitution of aluminum in the C–S–H gel
[15,16]	Prescription (use of a nano-modifying additive)	titanium nanoparticles	$TiO_{2}$ nanoparticles contribute to
[17,18]	Prescription (use of a nano-modifying additive)	carbon nanotubes, nanosilica	Seals cement seal and as a result increases the strength
[15,18,19]	Prescription (use of a nanomodifying additive)	carbon nanotubes	Nanosilica absorbs on the surface of carbon nanotubes and promotes the formation of hydration products on their surface, which improves adhesion between carbon nanotubes and cement matrix
[20]	Prescription (supplement use)	calcium sulfoaluminate cement and gypsum	Carbon nanotubes form a strong interfacial bond with cement matrices and also increase the proportion “of high-density calcium silicate hydrate (HD-CSH) gel compared to low-density CSH gel” [20]
[21]	Prescription (supplement use)	coal waste rock and slag powder	It is noted that the transition time of the gel is reduced with an increase in the number of additions of calcium sulfoaluminate and gypsum.

**Table 2:** Parameters of the composition of concrete mixtures.

Forming Technology	Consumption of Materials per 1 m^3^ of Concrete, kg			
	*C*, kg/m^3^	*W*, L/m^3^	*CS*, kg/m^3^	*S*, kg/m^3^
Vibration	380	175	1012	755
Centrifugation	400	180	1220	695
Vibrocentrifugation				

**Table 3:** Rheological characteristics of concrete mixtures.

Characteristic Name	Value	
	Vibration	Centrifugation/Vibrocentrifugation
Average density, kg/m^3^	2325	2467
Cone draft, cm	4	2
Solution separation of concrete mix, %	2.7	1.5
Water separation of concrete mix, %	0.3	0.1

**Table 4:** Results of experimental studies of the physical and mechanical characteristics of vibrated concrete.

Density ρ, kg/m^3^	Compressive Strength *R_b,cub_*, MPa	Axial Compressive strength *R_b_*, MPa	Axial Tensile Strength *R_bt_*, MPa	Tensile Strength in Bending *R_btb_*, MPa
2325 ± 37	49.7 ± 2.8	34.8 ± 1.9	3.15 ± 0.18	5.32 ± 0.31

**Table 5:** Integral strength characteristics.

Characteristic Name	Centrifuged Concrete	Vibro-Centrifuged Concrete
ρ, kg/m^3^	2459 ± 32	2472 ± 27
*R_b,cub_*, MPa	53.2 ± 2.7	62.7 ± 2.9
*R_b_*, MPa	37.8 ± 1.9	42.5 ± 2.0
*R_bt_*, MPa	3.84 ± 0.19	4.11 ± 0.20
*R_btb_*, MPa	6.31 ± 0.32	7.01 ± 0.29

**Table 6:** Difference in values (Δ_1_) of integral strength characteristics between vibrated, centrifuged, and vibrocentrifuged concretes.

Characteristics Name	Δ_1_, %		
	*V*	*C*	*VC* (Compared to *C*)
*R_b,cub_*, MPa	0	+7.0	+26.2 (+17.9)
*R_b_*, MPa	0	+8.6	+22.1 (+12.4)
*R_bt_*, MPa	0	+21.9	+30.5 (+7.0)
*R_btb_*, MPa	0	+18.6	+31.8 (+11.1)

**Table 7:** Difference in values (Δ_2_) of differential characteristics between centrifuged and vibrocentrifuged concretes.

Characteristics Name	Δ_2_, %					
	*C*			*VC*		
	Inner	Middle	Outer (Compared to Middle)	Inner	Middle	Outer (Compared to Middle)
*R_b,cub_*, MPa	0	+48.7	+117 (+45.8)	0	+70.1	+126 (+33.0)
*R_b_*, MPa	0	+64.8	+132 (+55.2)	0	+76.3	+130 (+30.3)
*R_bt_*, MPa	0	+55.0	+130 (+48.4)	0	+81.0	+133 (+28.9)
*R_btb_*, MPa	0	+65.6	+147 (+49.1)	0	+79.4	+144 (+36.1)

**Table 8:** Characteristics of Portland cement.

Characteristics	Value
Physical and mechanical characteristics of Portland cement	
Specific surface, m^2^/kg	352
Normal density of cement paste, %	26.0
Fineness of grinding, passage through a sieve No. 008, %	96.7
Setting time, min	
-start	165
-end	225
Tensile strength in bending, MPa:	
28 days	7.7
Compressive strength, MPa:	
28 days	56.2
Mineralogical composition of Portland cement	
C_3_S, %	68
C_2_S, %	13
C_3_A, %	7
C_4_AF, %	10

## Data Availability

The study did not report any data.
